# Modelling the Potential Population Impact and Cost-Effectiveness of Self-Testing for HIV: Evaluation of Data Requirements

**DOI:** 10.1007/s10461-014-0824-x

**Published:** 2014-06-24

**Authors:** Valentina Cambiano, Sue Napierala Mavedzenge, Andrew Phillips

**Affiliations:** 1 Research Department of Infection & Population Health, UCL, Rowland Hill Street, London, NW3 2PF UK; 2 Women’s Global Health Imperative, RTI International, San Francisco, CA USA

**Keywords:** HIV self-testing, Modelling, HIV, Cost-effectiveness, Data

## Abstract

HIV testing uptake has increased dramatically in recent years in resource limited settings. Nevertheless, over 50 % of the people living with HIV are still unaware of their status. HIV self-testing (HIVST) is a potential new approach to facilitate further uptake of testing which requires consideration, taking into account economic factors. Mathematical models and associated economic analysis can provide useful assistance in decision-making processes, offering insight, in this case, into the potential long-term impact at a population level and the price-point at which free or subsidized HIVST would be cost-effective in a given setting. However, models are based on assumptions, and if the required data are sparse or limited, this uncertainty will be reflected in the results from mathematical models. The aim of this paper is to describe the issues encountered in modeling the cost-effectiveness of introducing HIVST, to indicate the evidence needed to support various modeling assumptions, and thus which data on HIVST would be most beneficial to collect.

## Background

The scale up of antiretroviral therapy (ART) in resource limited settings (RLS) has transformed HIV from a terminal illness to a chronic condition. Nevertheless, many people living with HIV in need of ART still do not access it, or HIV care more broadly, because they are unaware of their HIV status. This results in increased morbidity and mortality [1, 2] and potentially higher risk behavior, as undiagnosed individuals may not have the motivation to reduce condomless sex that an HIV diagnosis can induce [3–6]. Importantly, low uptake of HIV testing will also limit effective implementation of new prevention strategies including male circumcision [7–9], vaginal, rectal and oral pre-exposure prophylaxis [10] and early ART [11]. Despite a dramatic increase in HIV testing in recent years in most sub-Saharan African countries, over 50 % of people living with HIV are unaware of their HIV status [12]. The reasons for not actively seeking an HIV test through current provider-delivered strategies (referred from now on as “HIV testing and counselling” (HTC), regardless of whether it is a client-initiated such as standard voluntary counselling and testing, or provider-initiated strategy, or of the location where it is performed) are numerous, including fear of stigma and discrimination, perceived lack of confidentiality, and the inconvenience and opportunity costs of testing [13]. Many of these barriers may be addressed through HIV self-testing (HIVST). HIVST consists of an individual collecting their own sample (typically saliva or a finger prick blood sample), and performing the HIV test on their own [14]. Though the idea of HIVST has been debated for over two decades, regulated HIVST kits are generally not available, with the United States and Kenya being among the few exceptions.

Given the imperative of expanding HIV testing uptake and frequency, it is important to consider new delivery strategies such as HIVST. The aim of this paper is to describe the issues encountered in modeling the potential effectiveness and cost-effectiveness of HIVST, to highlight what evidence exists to support assumptions, and to identify the areas where additional research is required in order to reduce uncertainty around these parameters. Ultimately, this will result in better-informed decision making around HIVST policy and programming.

## Rationale for Modeling HIV Self-Testing

Globally, donors and other stakeholders are considering whether investments should be made in the marketing and delivery of HIVST, in order to increase uptake of HIV testing. To maximize public health benefits, generally only cost-effective interventions should be introduced.

In RLS, preliminary research has been conducted in Kenya, Malawi and South Africa demonstrating high uptake, relatively good accuracy, and the potential to link self-testers to care [15–19]. However, we lack data on a number of other important factors including the increase in rate of first-time and repeat testing, the level of confirmatory testing, the long-term impact of introducing HIVST and its cost. Mathematical models can help us make best use of available data and provide insight into the potential impact of HIVST at a population level, including over the longer term, and can help determine whether the introduction of free or subsidized HIVST kits would be cost-effective. A summary of the issues involved in modelling HIVST is provided in Table 1.

**Table 1:** Summary of the issues highlighted and the data required

Issues:	Data required (including format of estimate)	Possible source of data (if available)
1. What proportion of population is resistant to HTC (i.e. they will be tested only if symptomatic)?	$\frac{{\# {\text{ Of people resistant to HTC}}}}{{\# {\text{ Of people in the entire population}}}}$	–
2. Would people who would not have accessed HTC opt to self-test?	$\frac{{\# \;{\text{Of people resistant to HTC who self-test}}}}{{\# {\text{ Of people resistant to HTC}}}}$	[17–19, 39]
3. Would people who would have accessed HTC to test for the first time instead opt for HIVST?	$\frac{{\# {\text{ Of first-time testers using HIVST}}}}{{\# {\text{ Of all first-time testers}}}}$	–
4. Would people who would have accessed HTC for repeat HIV testing instead opt for HIVST?	$\frac{{\# {\text{ Of HIVST conducted in people who tested before}}}}{{\# {\text{ Of tests }}\left( {\text{HIVST or HTC}} \right){\text{ conducted among people who tested for HIV before}}}}$	[16, 33, 43–46]
5. Is the subgroup of those who choose HIVST different from the population who access HTC?	Characteristics (including demographics and sexual behaviour) of people who choose HIVST, who choose HTC and who choose not to test for HIV	–
6. Would the availability of HIVST Increase the chance that people who have never tested (who are non-resistant to testing) of testing for the first time? If so, by how much?	$\frac{{\# {\text{ Of first-time testers}},{\text{ when HIVST is available}}}}{{\# {\text{ Of first-time testers}},{\text{ when HIVST is not available}}}}$ Data on total # of HTC administered and HIVST kits distributed, if possible broken down by whether first or repeat test, could help inform these parameters	–
7. Would the availability of HIVST increase the frequency of repeat testing? If so, by how much?	$\frac{\text{Rate of testing when HIVST is available}}{{{\text{Rate of testing when HIVST is not available}}}}$ Data on total # of HTC administered and HIVST kits distributed, if possible broken down by whether first or repeat tests, could help informing these parameters	[42]
8. Are the characteristics of people whose rate of testing increases with the availability of HIVST different from those for whom the frequency does not increase?	Data on characteristics of people with increased rate of testing and for whom the rate of testing does not increase (including demographics and sexual behaviour)	–
9. Do people seek confirmatory HTC following a positive HIVST?	$\frac{{\# {\text{ Of people who receive a positive HIVST and seek confirmatory HTC}}}}{{\# {\text{ Of people who receive a positive HIVST}}}}$	[15, 18, 39]
10. What is the sensitivity of HIVST when conducted by lay people?	$\frac{{\# {\text{ Of people with a positive result using HIVST and using the gold standard HTC}}}}{{\# {\text{ Of people with a positive result using gold standard HTC}}}}$	[16, 19, 33, 49, 50, 59–62]
11. What is the specificity of HIVST when conducted by lay people?	$\frac{{\# {\text{ Of people with a negative result using HIVST and using the gold standard HTC}}}}{{\# {\text{ Of people with a negative result using gold standard HTC}}}}$	[16, 19, 33, 49, 50, 59–62]
12. What, if any, change in sexual behaviour occurs following a positive and negative HIVST?	$\frac{{\#{\text{ Of condom-less partners or condom-less sex acts after a positive HIVST result}}}}{{\#{\text{ Of condom-less partners or condom-less sex acts before HIVST}}}}$ $\frac{{\# {\text{ Of condom-less partners or condom-less sex acts after a negative HIVST result}}}}{{ \# {\text{ Of condom-less partners or condom-less sex acts before HIVST }}\left( {{\text{and how this differs}},{\text{ if at all}},{\text{ from change after HTC}}} \right)}}$	[38]
13. What is the cost of implementing HIVST in RLS?	Cost of implementing a HIVST program if self-test kits are free to the end user (including kit and distribution and support costs)	–
14. What is the quality of life following a positive or a negative HIVST as compared to the same result communicated by a provider?	$\frac{\text{Quality of life after reading a positive HIVST result}}{\text{Quality of life before HIVST}}$ $\frac{\text{Quality of life after reading a negative HIVST result}}{\text{Quality of life before HIVST}}$ To compare against: $\frac{\text{Quality of life after a positive result communicated by a health care provider}}{\text{Quality of life before HTC}}$ $\frac{\text{Quality of life after a negative result communicated by a health care provider}}{{{\text{Quality of life before HTC }}}}$	[39]
15. How well do people link to post-test care after being diagnosed HIV-positive following HIVST, as compared to being diagnosed following HTC?	$\frac{{\# {\text{ Of people diagnosed with HIV who link to care }}\left( {\text{defined as evaluation of eligibility for ART initiation}} \right){\text{ following a positive HIVST}}}}{{\# {\text{ Of people who are diagnosed }}\left( {\text{have a confirmatory HTC}} \right){\text{ following a positive HIVST}}}}$ To compare against: $\frac{{\# {\text{ Of people diagnosed with HIV who link to care following a diagnosis not as a consequence of a positive HIVST}}}}{{\# {\text{ Of people who are diagnosed with HIV not as a consequence of a positive HIVST}}}}$	[18, 39]
16. How well are people retained in care after being diagnosed HIV-positive following HIVST, as compared to being diagnosed following HTC?	$\frac{{\# {\text{ Of people who are retained in care }}\left( {\text{among those eligible and ineligible for ART}} \right){\text{ at 1 and n years since diagnosis prompted by a positive HIVST}}}}{{\# {\text{ Of people who were diagnosed as a consequence of a positive HIVST 1 and n years ago}}}}$ To compare against: $\frac{{\# {\text{ Of people who are retained in care }}\left( {\text{among those eligible and ineligible for ART}} \right){\text{ at 1 and n years since diagnosis not prompted by a positive HIVST}}}}{{\# {\text{ Of people who were diagnosed with HIV}},{\text{ not as a consequence of a positive HIVST}},{\text{ 1 and n years ago}}}}$	–
Other considerations (which cannot be captured by the current model and have not been discussed due to the limited space)		
17. For people who have never tested for HIV, or do not attend regular repeat testing, what are the reasons for not testing? Which barriers, if any, might be overcome with the availability of HIVST? How can HIVST programming be designed to help overcome these barriers?	Some reasons for not having tested before: -Not wanting to know the result of the HIV test -Inconvenience/opportunity cost including: waiting time to have an HIV test, distance to the facility which provide HIV test, cost -Real or perceived lack of confidentiality -Stigma associated with testing -Not believing oneself to be at risk -Poor treatment by testing staff	[13]
18. Would people use HIVST to test new partners for HIV, and would they act on these results (by either using protection or not having sex)?	$\frac {{\text{ People who would use HIVST to test new partners}}}{\text{ People with new partners}}$	[32]

Several mathematical models have evaluated the impact and/or cost-effectiveness of expanding HTC in some high income [20–25] as well as low and middle income countries [26–29]. All found that HTC could be cost-effective in some circumstances, although at different frequencies and at different cost per quality adjusted life-year gained or disability adjusted life-year averted.Factors that have been found to influence the cost-effectiveness of HTC include: HIV incidence [28], HIV prevalence [26], prevalence of undiagnosed HIV infection [23], whether key populations were targeted [25], HTC cost [28], ART costs [28], and whether averting tertiary infections was taken into account [28].HIVST could lead to increased HIV testing, but to evaluate its effectiveness and cost-effectiveness it is necessary to take into consideration differences between HIVST and HTC.

Just one modeling study of HIVST has been conducted, among men having sex with men (MSM) in Seattle, USA. This study assessed the impact on HIV prevalence of replacing clinic-based HTC with HIVST [30]. The authors concluded that any replacement of clinic-based HTC with HIVST would increase HIV prevalence due to the longer “window period” (i.e. the time between initial HIV infection and when the test can reliably detect the infection) of the HIVST as compared to antigen–antibody combination tests used in some clinics [30]. However, currently in RLS antibody–only tests are also used by providers.To our knowledge, the cost-effectiveness of introducing HIVST in RLS, using a mathematical model, has not yet been evaluated.

## A Modeler’s ‘Wish List’ for HIV Self-Testing

Building a model requires a detailed hypothesis for how a process—in this case the introduction of HIVST—could work and which parameters need to be defined, and if possible estimated from data. The parameters characterized by high levels of uncertainty are usually varied in sensitivity analysis to understand how they affect the results and therefore what the impact might be of a wrong assumption.

**Fig. 1:**
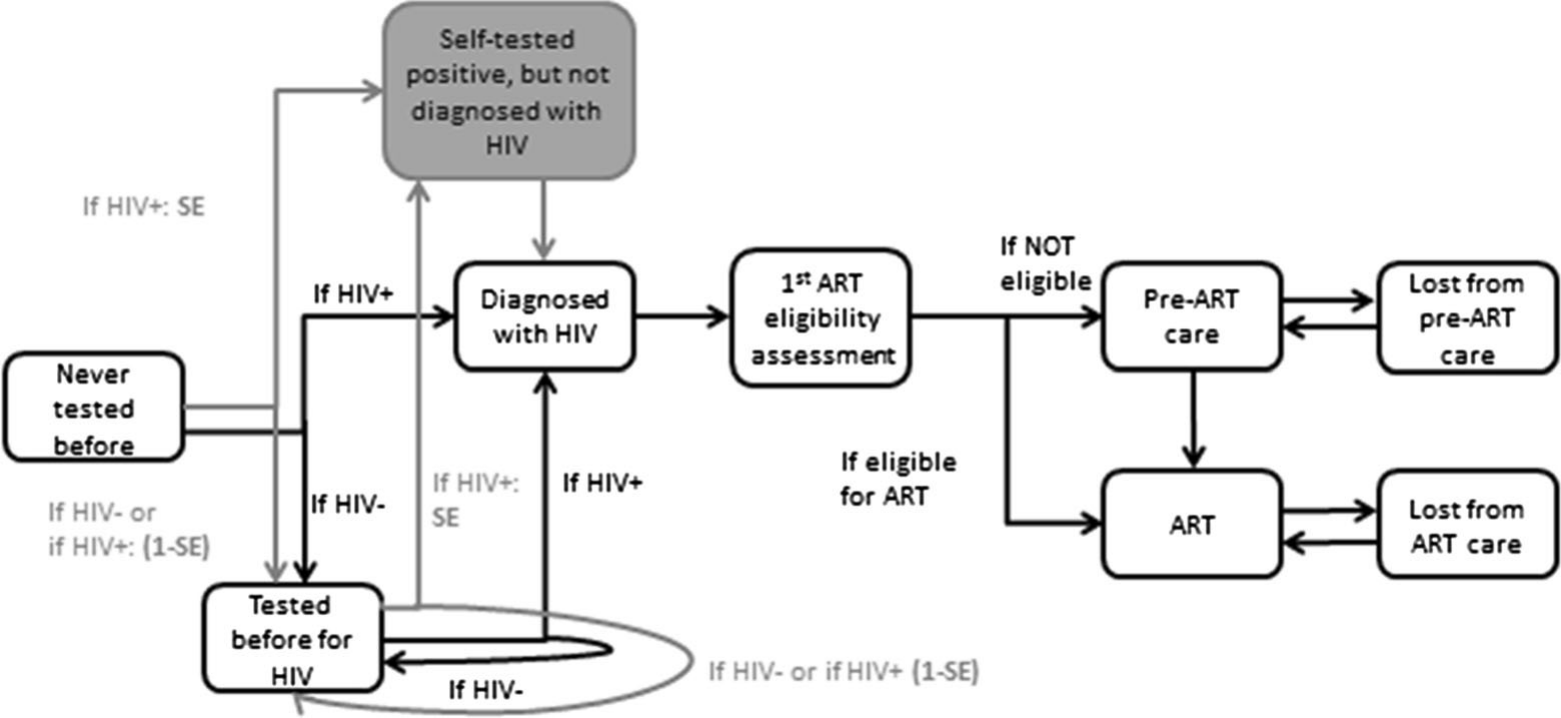
*Example of parameterization of self-testing in a mathematical model (Synthesis model* [57, 58]*)*. Illustration of features of a model incorporating HIVST—the section of the graph in grey only applies if HIVST is introduced. Features such as the level of sexual behaviour and whether the person is truly HIV infected would be included in such a model but are left cut here for the purposes of simplification. People can be tested for the first time using a provider delivered HTC at a different rate (in the model this rate may depend on sexual behaviour, age, gender, presence of symptoms and other factors). If HIV negative they respectively move or remain in the group of those who tested before for HIV, while if HIV positive are considered diagnosed with HIV. At this stage they experience a certain rate of having the 1st ART eligibility assessment, and once this is completed of being enrolled into pre-ART care, if not eligible for ART, or to be initiated on ART if eligible. Whether in pre-ART care or on ART, they can be lost from care and return back into care. If a person with HIV is self-tested for HIV, they have a chance equal to the sensitivity of the self-test (SE) that the result of the test is positive. If the test result is positive the person is not considered diagnosed with HIV, but there is a certain rate with which they will have a confirmatory provider-delivered HTC. For simplicity of illustration in this graph we have assumed that the test provider by a trained person is 100 % accurate and that the specificity of the self-test is 1. If a person has a self-test and either is not infected with HIV or, if HIV+, with a probability of (1−SE) the person would remain in the group of those who tested before for HIV. People with HIV diagnosed via self testing positive then follow the same path as those who tested for HIV using provider delivered HTC, although the rate at which they have the 1st ART eligibility assessment could differ. At these different stages the risk of morbidity and mortality and of infecting other people [not illustrated in the Figure) varies In the Synthesis model the risk of morbidity and mortality depends on age and gender and for people HIV-positive as well on CM-count, viral load, ART and PCP prophylaxis; while the risk of transmission mainly on the HIV-RNA level of the partner the person has condom-less sex with (Further details are available in [53, 54]).

Mathematical models designed to evaluate HIVST need to be dynamic and to consider testing in HIV positive and HIV negative people, levels of sexual risk behavior, and model the risk of acquiring HIV as a function of sexual risk behavior. Models also need to distinguish between HTC, which if positive implies diagnosis, and HIVST, which would require the additional step of confirmatory HTC before a person is considered diagnosed. A simple illustration of how HIVST could be included in a mathematical model is provided (Fig. 1).

### Proportion of People who do not Test Under Provider-Delivered Testing Strategies(Issue 1 in Table 1)

Although acceptability of HTC is generally high, there is substantial variability in uptake [31]. Furthermore, testing uptake varies considerably based on socio-demographic characteristics [12]. It is therefore necessary to define a proportion of the population as ‘resistant to testing’, meaning they would not utilize HTC.

### Uptake of Self-Testing Among Those Resistant to Testing (Issue 2 in Table 1)

It is necessary to quantify how many people might self-test from among those who are resistant to testing by existing available means. Several studies have evaluated hypothetical acceptability of HIVST [16, 32–37], while fewer have evaluated actual uptake when made available, and in particular among people who would not have accepted HTC [16, 17, 19, 38–40]. In a study conducted in Malawi, where HIVST was made available through community counsellors, 76 % of over 16,000 residents had used HIVST at 12 months since its introduction, with 43 % being first time testers [39]. A study conducted among healthcare workers in 7 hospitals in Kenya found that the uptake was 89 %. Among healthcare workers who self-tested, most (92 %) had tested before, and from these data we cannot estimate how many of those who accepted the HIVST were resistant to HTC [17]. Similarly a pilot study conducted among healthcare workers in Cape Town, reported a 93 % uptake of unsupervised HIVST [19], with 13 % being first time testers.

Other studies have evaluated the uptake of HIVST, without distinguishing whether these people were resistant to HTC or not, nor on whether they were first-time or repeat tests [38]. A recent meta-analysis, for example, estimated uptake of HIVST to be 87 % [41].

### Impact of Self-Testing Availability on the Rate of HIV Testing Among Those not Resistant to Testing (Issue 3, 6 and 7 in Table 1)

We are also interested in the extent to which availability of HIVST would, by providing a convenient and confidential option, increase the probability of testing in people who are not resistant to HTC but have never been tested before; similarly, we are interested in the frequency of repeat HIVST. To our knowledge the only relevant information available comes from a study among MSM in Australia, which found that 66 %reported that they would test more often if HIVST were available [42].

### Replacement of Provider-Delivered Testing with Self-Testing (Issue 4 in Table 1)

A further important consideration is what proportion of future tests via HTC would be conducted via HIVST if available. Again, this might be different for first time versus repeat testing and will depend on the cost of different strategies. It may be cost-saving to introduce HIVST if its provision is less expensive than HTC, despite its lower sensitivity and the necessity for confirmatory HTC for those self-testing positive. It is therefore important to quantify the extent and characteristics of those who replace HTC with HIVST, particularly regarding sexual behaviour. The only research which provides some indication of this parameter is reported preferences for HIVST versus HTC methods, and these data are largely from high income countries [16, 33, 43–46]. We may not be able to accurately estimate this parameter until HIVST is more widely available.

### Characteristics of People who opt for Self-Testing (Issue 5 and 8 in Table 1)

One of the advantages of HIVST is the potential for increased confidentiality, which could appeal to marginalized groups who are often more affected by stigma and discrimination, such as sex workers and MSM. To our knowledge there are no available data on the uptake of HIVST in these key groups. Mathematical models are a simplification of reality, so they would not include all variables which characterize these groups: ours for example could incorporate dependence on age, gender and sexual behaviour while other models may include specific subgroups, characterized by different routes of HIV transmission.

This parameter is particularly important because if HIVST availability encourages testing in those resistant to testing, and who are at increased risk of HIV, this could impact their risk behaviour and/or their infectiousness (if they receive ART), and therefore potentially reduce the number of new infections they contribute to.

### Accuracy of Self-Testing (Issue 10 and 11 in Table 1)

Poor accuracy of HIVST has long been a concern [47]. The Food and Drug Administration approved OraQuick in-Home HIV test kit has over 99 % sensitivity and specificity when conducted and interpreted by trained providers [48, 49], and when conducted by lay people but read by a provider [34]. When conducted and interpreted by lay people specificity remains over 99 % [16, 19, 49, 50], while sensitivity varies from 66.7 % (95 % CI: 30.9-91.0), reported in a small pilot study of unsupervised HIVST conducted among health care workers in South Africa, up to over 99 % [16, 19, 50, 51]. Minor procedural errors and request for extra help have been reported in a small proportion (10 %) of populations evaluated [16]. There are several studies on-going evaluating the accuracy of kits for HIVST by lay people.

### Confirmatory Testing Following a Positive Self-Test (Issue 9 in Table 1)

A crucial modelling parameter is how many of those who test HIV positive by HIVST have a subsequent confirmatory HTC, which can allow them to be formally diagnosed and to initiate linkage to care and treatment. This parameter is difficult to measure given the private nature inherent to HIVST. Some indirect indication comes from a cluster randomized trial conducted in Malawi evaluating home-based assessment and initiation of ART in the context of HIVST [15]. They found that the offer of home-based assessment and initiation of HIV care significantly increased the willingness to report positive self-test results, and led to a three-fold rate of ART initiation in the first 6 months as compared to receiving facility-based HIV care alone [15, 18], suggesting that without such home-based assessment a high proportion of people who self-test positive may not present for confirmatory HTC. These findings potential underestimate the proportion of people who would link to care, due to the short observation period and the fact that the measurement of linkage to care was based on the individual informing the clinic of having had a positive HIVST. More recent data from this study [39] indicate that 89 % of used HIVST kits distributed were returned. Analysis of results of these used kits suggests that 75 % of positive results were disclosed to a counsellor. If this disclosure meant they had a confirmatory HTC this would suggest around 75 % of positives were ‘diagnosed’.

### “Linkage and Retention in Care” Following a Reactive Self-Test Result (Issue 15 and 16 in Table 1)

A further concern is the possibility for low levels of linkage into care and treatment after HIVST, as well as, differential retention in care after confirmatory HTC (i.e. the person is truly diagnosed, from the health system perspective). This is an issue providers already struggle with under the current HIV testing strategies. If the population choosing to self-test is fundamentally different from those who choose HTC (for example, more marginalized), it is possible that linkage and retention in care after diagnosis may be more challenging.

In the control arm of the Malawi cluster randomized trial, despite a dramatic increase in the number of people testing for HIV with the introduction of HIVST, the proportion who linked to care was similar to that in the background population where only HTC was available [18]. One year after HIVST and home assessment was made available, 78 % of those who disclosed their reactive HIVST result to counsellors linked to care [39]. There are no other data on this topic to the best of our knowledge, though research is currently underway.

### Psychological Impact of HIV Self-Testing (Issue 14 in Table 1)

The psychological impact of receiving a positive HIVST result without the immediate support of a counselor also needs consideration. This could have implications for cost-effectiveness, since a lower quality of life among a proportion of these individuals would be factored into measures of effectiveness (quality adjusted life-years or disability adjusted life-years). The few data available showed very little evidence of serious harm [39], and there are no comparisons to the psychological impact via other testing methods [52]. Further research on this topic is currently underway.

### Change in Sexual Behavior Following Self-Testing (Issue 12 in Table 1)

There is evidence that people who test HIV-positive through voluntary counselling and testing have reduced sexual risk behavior [6]. Among those who tested HIV-negative, there was no evidence of behavior change [6]. With provider-initiated testing and counselling [53], most studies, but not all, reported an increase in condom use in both people who tested HIV-negative and positive. To our knowledge the only data available on the potential change in risk behavior following HIVST come from a study among MSM, where they reported that the 10 people identified as HIV positive through HIVST did not have sexual intercourse after learning their result [38].

### Cost of Self-Testing (Issue 13)

A few studies have highlighted that cost is potentially a significant barrier to accessing HIVST, even in high income countries [34, 54, 55], given that where currently available they must be purchased, while HTC is generally free.

Cost is likely to be an even greater barrier to HIVST in RLS. Research studies so far have distributed them for free in order to evaluate the uptake of HIVST. The most widely available oral fluid–based test, OraQuick ADVANCE Rapid HIV 1/2, is among the most expensive of the leading rapid tests, costing around US$4 in RLS [56]. The method of distribution is likely to have a major impact on the cost of HIVST, as well as most of the other parameters discussed here. Distribution is therefore fundamental when considering the impact of the HIVST parameters described here.

## Conclusion

HIV self-testing has great potential to increase knowledge of HIV status in RLS, where over half of HIV-infected individuals are currently unaware of their status. However, the question remains as to whether the introduction of HIVST would be effective and cost-effective.

Mathematical modelling can help to answer this question, though field data is required in order to accurately estimate important model parameters. In this paper we have outlined the most important of these parameters, which include the level of replacement of HTC with HIVST, the increase in the rate of first-time and repeat testing, and the level of confirmatory testing and linkage to post-test care, among others (Table 1). The definition of these parameters reflects the way we have attempted to model HIVST, but different models could conceive and structure the way in which HIVST impacts the HIV epidemic in a different way. While some field data exist, there are several HIVST parameters for which evidence is limited. To increase the accuracy of our model it is necessary to collect more data from well-powered studies. If accurate mathematical models can be developed to inform effectiveness and cost-effectiveness, they will be an important tool to guide policy and programming around HIVST, and ultimately to increase knowledge of HIV status and reduce transmission in countries which need it most.
